# A neighborhood-regularization method leveraging multiview data for predicting the frequency of drug–side effects

**DOI:** 10.1093/bioinformatics/btad532

**Published:** 2023-08-30

**Authors:** Lin Wang, Chenhao Sun, Xianyu Xu, Jia Li, Wenjuan Zhang

**Affiliations:** College of Artificial Intelligence, Tianjin University of Science and Technology, No. 9, 13th Street, Tianjin Economic-Technological Development Area, Tianjin 300457, China; College of Artificial Intelligence, Tianjin University of Science and Technology, No. 9, 13th Street, Tianjin Economic-Technological Development Area, Tianjin 300457, China; College of Artificial Intelligence, Tianjin University of Science and Technology, No. 9, 13th Street, Tianjin Economic-Technological Development Area, Tianjin 300457, China; College of Artificial Intelligence, Tianjin University of Science and Technology, No. 9, 13th Street, Tianjin Economic-Technological Development Area, Tianjin 300457, China; College of General Education, Tianjin Foreign Studies University, No. 117, Machang Road, Hexi District, Tianjin 300204, China

## Abstract

**Motivation:**

A critical issue in drug benefit-risk assessment is to determine the frequency of side effects, which is performed by randomized controlled trails. Computationally predicted frequencies of drug side effects can be used to effectively guide the randomized controlled trails. However, it is more challenging to predict drug side effect frequencies, and thus only a few studies cope with this problem.

**Results:**

In this work, we propose a neighborhood-regularization method (NRFSE) that leverages multiview data on drugs and side effects to predict the frequency of side effects. First, we adopt a class-weighted non-negative matrix factorization to decompose the drug–side effect frequency matrix, in which Gaussian likelihood is used to model unknown drug–side effect pairs. Second, we design a multiview neighborhood regularization to integrate three drug attributes and two side effect attributes, respectively, which makes most similar drugs and most similar side effects have similar latent signatures. The regularization can adaptively determine the weights of different attributes. We conduct extensive experiments on one benchmark dataset, and NRFSE improves the prediction performance compared with five state-of-the-art approaches. Independent test set of post-marketing side effects further validate the effectiveness of NRFSE.

**Availability and implementation:**

Source code and datasets are available at https://github.com/linwang1982/NRFSE or https://codeocean.com/capsule/4741497/tree/v1.

## 1 Introduction

Benefit-risk assessment in drug evaluation runs through the whole life cycle of drugs and is an important step in clinical research and post-marketing evaluation of drugs (Eichler *et al.* 2008). Determining the frequency of side effects is crucial in benefit-risk assessment of drugs, which is mainly conducted through randomized controlled trails (Concato *et al.* 2000). Compared with the limitations of high cost, long cycle, and difficult implementation for controlled trails, computational prediction of frequencies of side effects can aid to guide the controlled trails. However, most existing computational methods focus on predicting the associations between drugs and side effects, but not the frequencies of side effects (Cami *et al.* 2011, Wang *et al.* 2016, Ding *et al.* 2019, Nguyen *et al.* 2019, Cakir *et al.* 2021, Qian *et al.* 2022).

Based on the drug–side effect frequency matrix, Galeano *et al.* (2020) firstly proposed a non-negative matrix factorization (NMF) model for predicting frequencies of drug side effects. Zhao *et al.* (2021) used a graph attention model to extract multiview representations of drugs and side effects from a drug–side effect interaction graph. Multiview refers to the use of multiple different views to represent the same dataset, and each view can provide different data characteristics, so that the characteristics and laws of the data can be described more comprehensively. Zhao *et al.* (2022) constructed a multitask model for drug–side effect association and frequency prediction by integrating multiple similarities of drugs and side effects and using a deep learning framework. Xu *et al.* (2022) proposed a graph attention network model for predicting the frequencies of drug side effects, which is characterized by learning drug representations from molecular graphs and using matrix factorization as a decoder. Among these methods, Zhao *et al.* (2021, 2022) did not fully consider the sparsity of available drug–side effect frequency terms and the interval between distributions of predicted scores of unknown and known frequency terms, resulting their models being prone to predict most unknown frequency terms as frequency terms.

**Figure 1.**
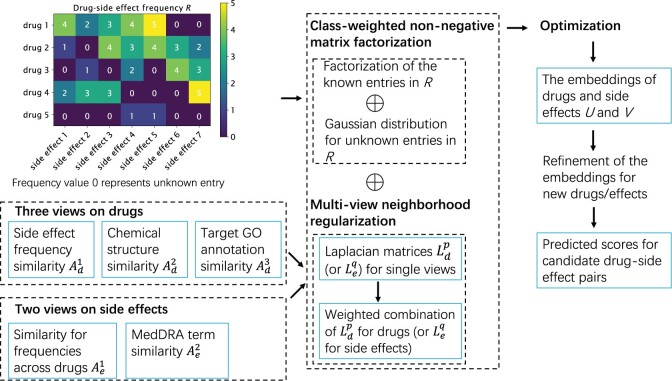
Flowchart of the drug–side effect frequency prediction method NRFSE.

The NMF model proposed by Galeano *et al.* (2020) has shown some advantages in terms of predictive performance and interpretability. However, it did not fully utilize the multiview data of drugs and side effects, so its prediction performance could be further improved. In this work, we improve the NMF model and propose a neighborhood-regularization method (NRFSE) for predicting frequencies of drug side effects. Figure 1 shows the flowchart that illustrates the main components of the proposed NRFSE method. Our contributions of this work are as follows. First, we decompose the drug–side effect frequency matrix by using a class-weighted NMF, in which a Gaussian distribution is adopted to characterize the predicted scores of unknown drug–side effect pairs. Second, three views on drugs, including side effect frequency, chemical structure, and Gene Ontology (GO) annotations of drug targets, and two views of side effects, including frequencies across drugs and Medical Dictionary for Regulatory Activities (MedDRA) terms are integrated by multiview neighborhood regularization. Specifically, the Laplacian matrix for the multiview neighborhood is constructed by the weighted combination of the Laplacian matrices for the neighborhoods of single views. The regularization on multiview neighborhood is conducted so that most similar drugs and most similar side effects have similar embeddings, and the optimal weight for each view is adaptively determined. At last, the embeddings of new drugs and new side effects is not accurate, and we refine their embeddings by using the embeddings of their neighbors. To demonstrate the effectiveness of our method NRFSE, we applied it to one benchmark dataset and compared it with five state-of-the-art methods using 10-fold cross-validation (CV). The results showed that the prediction performance of NRFSE significantly outperformed the other methods. Furthermore, an independent test set demonstrated the utility of NRFSE for identifying correct frequencies of post-marketing side effects.

## 2 Materials and methods

### 2.1 Dataset

The drug–side effect frequency data used in this study was derived from Galeano *et al.* (2020) and Xu *et al.* (2022), which contains 750 drugs and 994 side effects. Then we obtained drug targets for each drug from DrugBank (Wishart *et al.* 2018) and removed drugs with no target information. Afterward, the final dataset includes 664 drugs and 994 side effects. The dataset takes drug–side effect frequency items from Side Effect Resource (SIDER 4.1, Kuhn *et al.* 2016) and encodes them with integers into five categories: very rare (frequency = 1), rare (frequency = 2), not frequent (frequency = 3), frequent (frequency = 4), and very frequent (frequency = 5). We use a frequency matrix *R* of dimensions 664 × 994 to represent the frequency relationships between drugs and effects, where non-zero values indicate known frequency items and 0 otherwise. The frequency matrix *R* contains 34 604 known frequency items, which only account for 5.24% of all the elements of the matrix (664 × 996). It is worth noting that among all non-zero items, the proportions of very rare, rare, infrequent, frequent, and very frequent items are 3.16%, 11.83%, 27.56%, 46.84%, and 10.87%, respectively.

We downloaded Simplified Molecular Input Line Entry Specification (SMILES) representations of drugs from PubChem (Wang *et al.* 2009) and obtained GO annotations of drug targets from DrugBank. MedDRA terminologies of side effects were derived from Adverse Drug Reaction Classification System (ADReCS, Cai *et al.* 2015) database. MedDRA is assigned and associated through a five-level structure, namely System Organ Class (SOC), High Level Group Term (HLGT), High Level Term (HLT), Preferred Term (PT), and Lowest Level Term (LLT), which vary from very general to very specific. We chose the information from the first four levels, i.e. SOC, HLGT, HLT, and PT, to encode side effects.

### 2.2 Various similarity measures

For any pair of drugs, we computed frequency similarity of their side effects, chemical structure similarity, and GO annotation similarity of their targets. Specifically, effect frequency similarity of any two drugs was calculated as the cosine similarity between their corresponding side effect frequency profiles (the two rows in *R*), and consequently a similarity matrix for drugs $A_{d}^{1}\in R^{n\times n}$ was generated, where *n *=* *664 is the number of drugs. We used Python’s open source toolkit for cheminformatics RDKit to convert SMILES representations of drugs into molecular graphs, and then calculated the chemical structure similarity between any two drugs based on topological fingerprints and Tanimoto similarity measure. In this way we can generate a structure similarity matrix $A_{d}^{2}\in R^{n\times n}$. GO covers three aspects of biology: cellular component (CC), molecular function (MF), and biological process (BP). Here we used MF to represent GO information of drug targets. For each drug, we encoded the GO information of all its targets as a multi-hot vector. Then we calculated GO similarity between any two drugs by using Jaccard coefficient and obtained a GO similarity matrix $A_{d}^{3}\in R^{n\times n}$.

For any pair of side effects, we computed frequency similarity of their associated drugs and hierarchical similarity of their MedDRA terms. Specifically, for any two effects, the cosine similarity of their corresponding frequency profiles (the two columns in *R*) was calculated, and then a similarity matrix for side effects $A_{e}^{1}\in R^{m\times m}$ was generated, where *m *=* *994 is the number of side effects. As to MedDRA, we chose the information from the first four levels, i.e. SOC, HLGT, HLT, and PT, to encode side effects. For each side effect, its MedDRA terms were encoded as a multi-hot vector and Jaccard coefficient was used to compute the hierarchical similarity between any two side effects. In this way, we obtained a hierarchical similarity matrix $A_{e}^{2}\in R^{m\times m}$.

### 2.3 Class-weighted non-negative matrix factorization

The frequency matrix *R* is decomposed into the drug feature matrix $U\in R^{n\times k}$ and the side effect feature matrix $V\in R^{k\times m}$, where *k* is the latent feature dimension of drugs and side effects, and *U* and *V* are both non-negative. The entries of *U* and *V* are all non-negative and hence only non-subtractive basis combinations are allowed. For each drug *d_i_* (i=1,2,…,n), the *i*-th row *u_i_* in *U* represents the latent signature of *d_i_*. Similarly, for each side effect *e_j_* (j=1,2,…,m), the *j*-th column *v_j_* in *V* represents the latent signature of *e_j_*. Formally, the any known drug–side effect frequency *R_ij_* (> 0) can be approximated by the inner product of *u_i_* and *v_j_* as follows:


(1) $$\min\frac{1}{2}∥I^{\Omega }⊙\left(R-UV\right){∥}_{F}^{2}$$

(2) s.t. U≥0,V≥0,

where ⊙ is the Hadamard product,$∥.{∥}_{F}$ is the Frobenius norm, and $I^{\Omega }$ is the mapping function, i.e. $I_{ij}^{\Omega }=1$ if *R_ij_* > 0 and 0 otherwise.

Since *R* is very sparse, for the unknown drug–side effect pairs (*R_ij_* = 0), we assume that their predicted scores ${\left(UV\right)}_{ij}$ satisfy a Gaussian distribution with mean *μ* and variance $\sigma^{2}$, i.e. $P({\left(UV\right)}_{ij}|R_{ij}=0)=N(\mu ,\sigma^{2})$. The log-likelihood function is $-\frac{1}{2\sigma^{2}}\sum_{R_{ij}=0}{\left({\left(UV\right)}_{ij}-\mu \right)}^{2}+C$. Combining Formulas (1 and 2), the model becomes as follows:


(3) $$\min\frac{1}{2}∥I^{\Omega }⊙\left(R-UV\right){∥}_{F}^{2}+\frac{\alpha }{2}∥I^{o}⊙\left(UV-\mu E\right){∥}_{F}^{2}$$

(4) s.t. U≥0,V≥0,

where $\alpha =1/\sigma^{2}$, *E* is a matrix with all ones, and *I^o^* is the mapping function, i.e. $I_{ij}^{o}=1$ if *R_ij_* = 0 and 0 otherwise.

### 2.4 Multiview neighborhood regularization

Our model is based on the assumption that “similar drugs may have similar side effects, and similar side effects may be caused by similar drugs”, so we expect similar drugs or effects to have similar latent signatures. Considering the multiview similarity for drugs and side effects, we propose a multiview NRFSE to regularize the latent features of drugs or side effects.

For the similarity data $A_{d}^{p}$ (*p *=* *1, 2, 3) of each view of the drug, drug pairs with lower similarity will lead to the introduction of noise. In this study, neighborhood regularization is adopted, that is, only *k*_1_ nearest neighbor drugs are considered, so that for drug pairs with greater similarity, their embeddings in the latent space are more similar (Liu *et al.* 2016, Zhang *et al.* 2020). After obtaining the *k*_1_ nearest neighbor information of the drug, we update the similarity matrix of each view to:


(5) $${\tilde{A}}_{d}^{p}\left(i,\mu \right)=\{\begin{array}{cc} A_{d}^{p}\left(i,\mu \right) & \;\text{if}\;d_{\mu }\in N^{p}\left(d_{i}\right)\backslash d_{i}\\ 0 & \;\text{otherwise}\; \end{array},$$

where $N^{p}\left(d_{i}\right)\backslash d_{i}$ is the *k*_1_-nearest neighbors of drug *d_i_*, ${\tilde{A}}_{d}^{p}$ is the updated similarity matrix and asymmetric. The neighborhood regularization for a single view is as follows:


(6) $$\min\frac{1}{2}\sum_{i=1}^{n}\sum_{\mu =1}^{n}{\tilde{A}}_{d}^{p}\left(i,\mu \right)∥u_{i}-u_{\mu }{∥}_{F}^{2}=\frac{1}{2}tr\left(U^{T}L_{d}^{p}U\right),$$

where tr() means the trace of a matrix, Laplacian matrix $L_{d}^{p}=\left(D_{d}^{p}+{\tilde{D}}_{d}^{p}\right)-\left({\tilde{A}}_{d}^{p}+{\tilde{A}}_{d}^{p}{}^{T}\right),\;D_{d}^{p}$ and ${\tilde{D}}_{d}^{p}$ are two diagonal matrices, $D_{d}^{p}(i,i)=\sum_{\mu =1}^{n}{\tilde{A}}_{d}^{p}\left(i,\mu \right),\;\;{\tilde{D}}_{d}^{p}(\mu ,\mu )=\sum_{i=1}^{n}{\tilde{A}}_{d}^{p}\left(i,\mu \right)$.

To fully utilize the multiview data for drugs, Laplacian matrix for the multiview neighborhood is constructed by the weighted combination of the Laplacian matrices of single views. We propose the multiview neighborhood regularization as follows:


(7) $$\min\frac{1}{2}tr\left(U^{T}\sum_{p=1}^{x}w_{p}{}^{\delta }L_{d}^{p}U\right)$$

(8) $$s.t.\;\sum_{p=1}^{x}w_{p}=1,\;w_{p}\geq 0,$$

where *x *=* *3 is the number of drug views, *w_p_* is the weight for each drug view, and *δ* is the power of *w_p_*. Notably, we set *δ*  =  2 so that all the views can contribute to the prediction.

Similarly, for side effects, the neighborhood regularization of each view is described as follows:


(9) $$\min\frac{1}{2}tr\left(VL_{e}^{q}V^{T}\right),$$

where $L_{e}^{q}=\left(D_{e}^{q}+{\tilde{D}}_{e}^{q}\right)-\left({\tilde{A}}_{e}^{q}+{\tilde{A}}_{e}^{q}{}^{T}\right),\;D_{e}^{q}$ and ${\tilde{D}}_{e}^{q}$ are two diagonal matrices, ${\tilde{A}}_{e}^{q}\left(i,\mu \right)$ is $A_{e}^{q}\left(i,\mu \right)$ if $e_{\mu }\in N^{q}\left(e_{i}\right)\backslash e_{i}$, and is 0 otherwise, $D_{e}^{q}(i,i)=\sum_{\mu =1}^{m}{\tilde{A}}_{e}^{q}\left(i,\mu \right),\;{\tilde{D}}_{e}^{q}(\mu ,\mu )=\sum_{i=1}^{m}{\tilde{A}}_{e}^{q}\left(i,\mu \right)$. We propose the multiview neighborhood regularization for side effects as follows:


(10) $$\min\frac{1}{2}tr\left(V\sum_{q=1}^{y}h_{q}{}^{\delta }L_{e}^{q}V^{T}\right)$$

(11) $$s.t.\;\sum_{q=1}^{y}h_{q}=1,\;h_{q}\geq 0,$$

where *y *=* *2 is the number of effect views, *h_q_* is the weight for each effect view.

By integrating Formulas (3), (4), (7), (8), (10), and (11), we can conclude the final multiobjective optimization as follows:


(12) $$\begin{array}{c} \underset{U,V,w_{p},h_{q}}{\min}\frac{1}{2}∥I^{\Omega }⊙\left(R-UV\right){∥}_{F}^{2}+\frac{\alpha }{2}∥I^{o}⊙\left(UV-\mu E\right){∥}_{F}^{2}\\ +\frac{\beta }{2}tr\left(U^{T}\sum_{p=1}^{x}w_{p}{}^{\delta }L_{d}^{p}U\right)+\frac{\gamma }{2}tr\left(V\sum_{q=1}^{y}h_{q}{}^{\delta }L_{e}^{q}V^{T}\right) \end{array}$$

(13) s.t. U≥0,V≥0,

(14) $$\sum_{p=1}^{x}w_{p}=1,\;w_{p}\geq 0,$$

(15) $$\sum_{q=1}^{y}h_{q}=1,\;h_{q}\geq 0,$$

where *β* and *γ* are regularization coefficients for drugs and side effects, respectively.

### 2.5 Solving algorithm

We use an iterative update algorithm to solve our model. Specifically, we randomly initialize *U* and *V* and normalize them using the Frobenius norm. $w_{p}\;(p=1,\ldots ,x)$ and $h_{q}\;(q=1,\ldots ,y)$ are initialized as $\frac{1}{x}$ and $\frac{1}{y}$, respectively. Then we update them using the following formulas:


(16) $$\begin{array}{c} U\leftarrow U_{0}⊙\\ \frac{\left(R+\alpha \mu I^{o}\right)V_{0}^{T}+\beta \left(\sum_{p=1}^{x}w_{p}{}^{\delta }\left({\tilde{A}}_{d}^{p}+{\tilde{A}}_{d}^{p}{}^{T}\right)\right)U_{0}}{\left(I^{\Omega }⊙\left(U_{0}V_{0}\right)+\alpha I^{o}⊙\left(U_{0}V_{0}\right)\right)V_{0}^{T}+\beta \left(\sum_{p=1}^{x}w_{p}{}^{\delta }\left(D_{d}^{p}+{\tilde{D}}_{d}^{p}\right)\right)U_{0}}, \end{array}$$

(17) $$\begin{array}{c} V\leftarrow V_{0}⊙\\ \frac{U^{T}\left(R+\alpha \mu I^{o}\right)+\gamma V_{0}\left(\sum_{q=1}^{y}h_{q}{}^{\delta }\left({\tilde{A}}_{e}^{q}+{\tilde{A}}_{e}^{q}{}^{T}\right)\right)}{U^{T}\left(I^{\Omega }⊙\left(UV_{0}\right)+\alpha I^{o}⊙\left(UV_{0}\right)\right)+\gamma V_{0}\left(\sum_{q=1}^{y}h_{q}{}^{\delta }\left(D_{e}^{q}+{\tilde{D}}_{e}^{q}\right)\right)}, \end{array}$$

(18) $$w_{p}=\frac{\frac{1}{{tr{(U}^{T}L_{d}^{p}U)}^{\frac{1}{\delta -1}}}}{\sum_{p=1}^{x}\frac{1}{{tr{(U}^{T}L_{d}^{p}U)}^{\frac{1}{\delta -1}}}},$$

(19) $$h_{q}=\frac{\frac{1}{tr{\left(VL_{e}^{q}V^{T}\right)}^{\frac{1}{\delta -1}}}}{\sum_{q=1}^{y}\frac{1}{tr{\left(VL_{e}^{q}V^{T}\right)}^{\frac{1}{\delta -1}}}},$$

where *U*_0_ and *V*_0_ represent the matrices before updating, and *U* and *V* are the matrices after updating. We set the maximum number of iterations *max_iter_* = 500. Notably, for the new drug *d_i_* without any known side effect frequency items, i.e. the *i*-th row of *R* is a zero vector, the embedding *u_i_* of *d_i_* obtained by the above iterative process is not accurate. We replace *u_i_* with the weighted average of the embeddings of its *k*_2_ nearest neighboring (*k*_2_-NN) drugs having known side effects. Similarly, the embedding of the new side effect is replaced with the weighted average of the embeddings of its *k*_2_-NN known effects. The details of the optimization algorithm for solving Formulas (12–15) are shown in Algorithm 1 and Supplementary Material. As to the hyperparameter settings, we set *α *= 0.05, *β *= 2, *γ *= 2, *μ *= 1, *k *=* *200, *k*_1_=20, and *k*_2_=10 for warm-start scenario, and *α *= 0.05, *β *= 4, *γ *= 4, *μ *= 1, *k *=* *200, *k*_1_=10, and *k*_2_=10 for cold-start scenario, which achieve the best performances for our model.

**Algorithm 1** NRFSE

**Input:** input Drug–side effect frequency matrix *R*, similarity matrices for drugs $A_{d}^{1},\;A_{d}^{2}$ and $A_{d}^{3}$, similarity matrices for side effects $A_{e}^{1}$ and $A_{e}^{2}$;

**Output:** output result

1: Compute similarity matrices for *k*_1_-nearest neighbors ${\tilde{A}}_{d}^{p}$ and ${\tilde{A}}_{e}^{q}$, diagonal matrices $D_{d}^{p},\;{\tilde{D}}_{d}^{p},\;D_{e}^{q}$ and ${\tilde{D}}_{e}^{q}$, Laplacian matrices $L_{d}^{p}$ and $L_{e}^{q}\;(p=1,2,3,q=1,2)$;

2: Initialize *U*, *V*, *w_p_* and *h_q_*;

3: **for**  $\mathrm{iter}=1,\ldots ,max_{\mathrm{iter}}$  **do**

4:  Update *U*, *V*, *w_p_* and *h_q_* according to Formulas (16–19);

5: **end for**

6: **for** new drug *d_i_* **do**

7:  Set the embedding $u_{i}=\frac{\sum_{d_{j}\in N(d_{i})}s_{ij}^{d}u_{j}}{\sum_{d_{j}\in N(d_{i})}s_{ij}^{d}}$, where $N\left(d_{i}\right)$ represents the *k*_2_ nearest neighbors of known drugs sorted by $s_{ij}^{d},\;s_{ij}^{d}=\sum_{p=2}^{x}w_{p}{}^{\delta }{\tilde{A}}_{d}^{p}\left(i,j\right)$;

8: **end for**

9: **for** new side effect *e_j_* **do**

10:  Set the embedding $v_{j}=\frac{\sum_{e_{i}\in N(e_{j})}s_{ij}^{e}v_{i}}{\sum_{e_{i}\in N(e_{j})}s_{ij}^{e}}$, where $N\left(e_{j}\right)$ represents the *k*_2_ nearest neighbors of known effects sorted by $s_{ij}^{e},\;s_{ij}^{e}=\sum_{q=2}^{y}h_{q}{}^{\delta }{\tilde{A}}_{e}^{q}\left(i,j\right)$;

11: **end for**

12: **return** *UV*

### 2.6 Evaluation metrics

To evaluate the effectiveness of our model, we set up 10-fold CVs under the warm-start and cold-start scenarios, respectively. The warm-start scenario refers to the discovery of frequencies of novel side effects of a drug with some already available side effect frequencies. In the warm-start scenario, we randomly divide all known drug–side effect frequency entries into 10 folds, and set each fold as the test set in turn while the remaining 9 folds as the training set. We denote this CV setting as CV1.

The cold-start scenario for drugs means to predict frequencies of side effects of a new drug, which has no known side effects before. As to the cold-start scenario, we randomly divide all drugs into 10 folds, and in turn, the associated effect frequencies of each fold are deemed as the test set while the rest are used as the training set. We denote this CV setting as CV2. Notably, we set the frequency terms of the test set to 0 during the training process for both scenarios.

We evaluate the performance of the model in terms of drug–side effect association identification and frequency value prediction. On the one hand, we use the area under the receiver operating characteristic (ROC) curve (AUC) and the area under the Precision-Recall curve (AUPR) to assess the association identification performance. We compute metrics for each drug on the test set and then use their average as the performance metric for each fold. Specifically, for each drug in the test set, we take the frequency items of this drug in the test set as positive samples, and all the unknown frequency items of this drug as negative samples. On the other hand, for frequency prediction, we use root mean square error (RMSE), mean absolute error (MAE), and Pearson correlation coefficient (PCC) as evaluation metrics as follows:


(20) $$\mathrm{RMSE}=\sqrt{\frac{\sum_{i,j}{\left(P_{i,j}-R_{i,j}\right)}^{2}}{t}},$$

(21) $$\mathrm{MAE}=\frac{\sum_{i,j}\left|P_{i,j}-R_{i,j}\right|}{t},$$

(22) $$\mathrm{PCC}=\frac{\sum_{i,j}\left(P_{i,j}-\bar{P}\right)\left(R_{i,j}-\bar{R}\right)}{\sqrt{\sum_{ij}{\left(P_{i,j}-\bar{P}\right)}^{2}}\sqrt{\sum_{ij}{\left(R_{i,j}-\bar{R}\right)}^{2}}},$$

where *t* is the number of known drug–side effect frequency items in the test set for each fold, $P_{i,j}$ and $R_{i,j}$ are the predicted score and true frequency of the drug-effect pair (*d_i_*, *e_j_*), $\bar{P}$ and $\bar{R}$ are their averages. For AUC, AUPR, RMSE, MAE, and PCC, we obtain the average of the 10-fold results as the final performance score.

## 3 Results

### 3.1 Comparisons with baseline methods

To verify the performance of our model, we compared with four state-of-the-art methods for predicting frequencies of side effects, including Galeano’s model (Galeano *et al.* 2020), MGPred (Zhao *et al.* 2021), SDPred (Zhao *et al.* 2022), and DSGAT (Xu *et al.* 2022). We adopted their default optimal parameters for each method in the comparison experiments. IGMC (Zhang and Chen 2019) is an inductive matrix completion method based on graph neural network, and here we use IGMC for the completion of drug–side effect frequency matrix.

Our model NRFSE is compared with the above baseline methods under CV1 setting. Table 1 shows the comparison results obtained by all the methods. It is worth noting that Galeano’s model, DSGAT and NRFSE all use the frequency prediction scores to calculate AUC and AUPR. As to MGPred, the authors regarded frequency prediction and association prediction of drug–side effect items as two independent tasks and trained them separately, so that for the same drug–side effect items, the association scores and frequency scores were not consistent. Here, we use the frequency scores of MGPred to calculate AUC and AUPR for consistency of comparison methods. SDPred integrates association prediction and frequency prediction into one framework and obtains association scores and frequency scores simultaneously. The values of AUC and AUPR can be obtained based on association scores and frequency scores, respectively, and we denoted them by SDPred_A and SDPred_F. It can be seen that the AUC values of Galeano’s model, SDPred_A, DSGAT and NRFSE are >0.9, and the AUPR values of these methods are higher than 0.2. As a contrast, MGPred, SDPred_F, and IGMC have lower AUC and AUPR values. As for frequency prediction indicators such as RMSE, MAE, and PCC, the three methods, i.e. MGPred, SDPred_F, and IGMC, exhibit better performance. This indicates that although these three methods fit the known frequencies of drug side effects very well, they are prone to introduce false positives, that is, a lot of drug–side effect pairs of unknown associations are predicted to be associated. The above results limit the use of these three methods in clinical trials. SDPred_A can only predict drug–side effect associations, and its AUC and AUPR values are still lower than NRFSE. NRFSE achieves the best AUC and AUPR, and AUPR is 11.9% better than the second-ranked method DSGAT, while PCC is 4.5% higher than that of DSGAT.

**Table 1.** The comparison results under CV1 setting.

Method	AUC	AUPR	RMSE	MAE	PCC
Galeano’s model	0.907	0.216	1.298	0.953	0.478
MGPred	0.762	0.120	0.643	0.486	0.734
SDPred_F	0.583	0.077	**0.593**	**0.433**	**0.781**
SDPred_A	0.919	0.230	\	\	\
IGMC	0.760	0.123	0.616	0.454	0.758
DSGAT	0.917	0.243	1.031	0.754	0.557
NRFSE	**0.926**	**0.272**	1.008	0.767	0.582

‘\’ indicates that there is no corresponding result. The best results are in bold.

We conducted the comparison experiments under CV2 setting in the following. It is worth noting that Galeano’s model results in a prediction score of zero for each side effect of a new drug, so this method cannot be used for cold start scenario of drugs. Likewise, MGPred, SDPred_F, and IGMC obtain lower RMSE values, but their AUC and AUPR values are also worse (see Table 2). NRFSE has the best prediction performance as to AUC and AUPR. Regarding AUPR, NRFSE is 7.5% better than the second-ranked DSGAT, while PCC is 8.3% higher than that of DSGAT. Furthermore, we show the performance of NRFSE when controlling for chemical similarities between drugs. We filtered out the drugs in the test set whose chemical similarity (Tanimoto coefficient) to a drug in the training set is no <0.6. Consequently, the numbers of drugs in the test set for each fold are 34, 37, 30, 31, 38, 37, 33, 33, 34, and 29. The results of performance of NRFSE for the remaining drugs are AUC = 0.897, AUPR = 0.426, RMSE = 1.346, MAE = 1.112, and PCC = 0.476, which are comparable to those of unfiltered scenario.

**Table 2.** The comparison results under CV2 setting.

Method	AUC	AUPR	RMSE	MAE	PCC
Galeano’s model	\	\	\	\	\
MGPred	0.624	0.204	1.007	0.795	0.251
SDPred_F	0.728	0.193	**0.868**	**0.634**	**0.487**
SDPred_A	0.874	0.403	\	\	\
IGMC	0.725	0.187	0.903	0.693	0.440
DSGAT	0.881	0.411	1.436	1.150	0.434
NRFSE	**0.898**	**0.442**	1.378	1.142	0.470

‘\’ indicates that there is no corresponding result. The best results are in bold.

### 3.2 Independent test and interpretability

**Figure 2.**
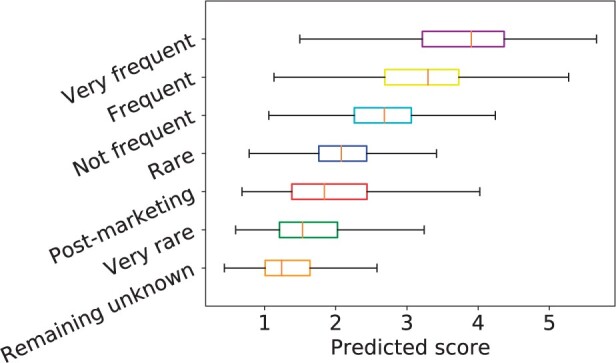
Predicted scores for side effects of different frequency categories obtained by NRFSE.

We obtained 8678 post-marketing side effects for these 664 drugs from SIDER. These post-marketing side effects are generally considered to be very rare because they have not been detected in clinical trials, while were reported after the drug entered the market. Notably, the available side effects in the frequency matrix *R* did not include these post-marketing effects. We used all known entries (frequency≥1) in *R* as the training set and predicted the frequency values of all unknown entries (frequency = 0). Then these unknown entries were divided into two parts, namely, the post-marketing side effects and the remaining unknown drug-effect associations. Figure 2 shows that most of the frequency predictive scores of post-marketing effects (median = 1.839) are between the predictive scores of very rare and rare categories (median is 1.534 and 2.078 respectively), and the difference between the predictive scores of the remaining unknown drug-effect associations and those of the very rare category is significant ($P=3.351\times 10^{-65}$, two-sided Wilcoxon rank-sum test). It is worth noting that the predictive scores of very rare, rare, not frequent, frequent, and very frequent categories were obtained by 10-fold CV under CV1 setting. The results illustrate that NRFSE can identify potential side effects from unknown associations and can accurately predict the frequency of these post-marketing side effects.

**Figure 3.**
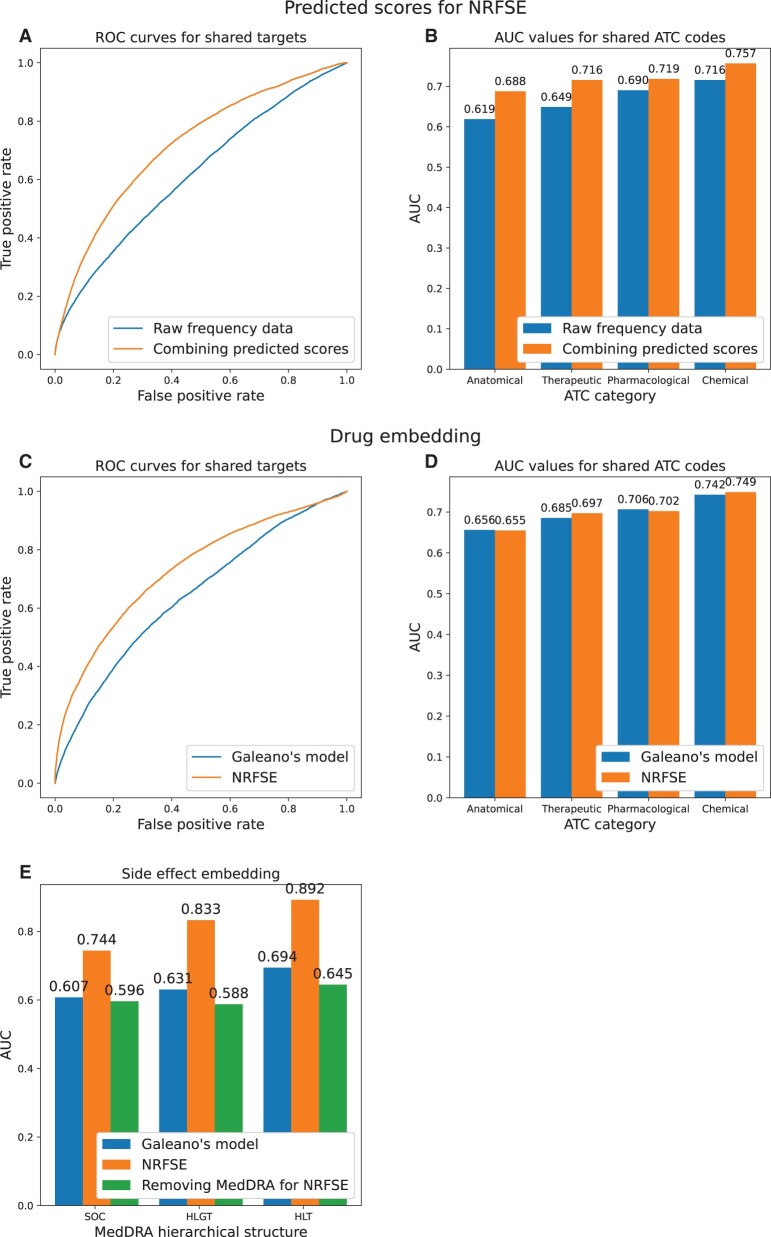
Interpretability of the prediction results of NRFSE. (A) The ROC curves for raw frequency data (AUC = 0.619) and that combining predicted scores (AUC = 0.719) as to shared drug targets. (B) The AUC bar charts for raw frequency data and that combining predicted scores as to shared ATC codes. (C) The ROC curves for drug embeddings obtained from Galeano’s model (AUC = 0.642) and NRFSE (AUC = 0.732) as to shared drug targets. (D) The AUC bar charts for drug embeddings obtained from Galeano’s model and NRFSE as to shared ATC codes. (E) The AUC bar charts for side effect embeddings obtained from Galeano’s model, NRFSE, and NRFSE without MedDRA information.

Then, we analyzed the interpretability of the prediction results. We replaced the unknown entries in *R* with the predicted frequency scores, and got a matrix denoted as $\hat{R}$. Based on the side effect profiles of *R* or $\hat{R}$, the cosine similarity was used to calculate the side effect similarity between any two drugs. Then, according to whether the two drugs share a target, drug pairs were labeled as positive or negative samples, whose numbers are 7149 and 21 2967, respectively. Figure 3A shows the ROC curves for *R* and $\hat{R}$, respectively, where the AUC values are 0.619 and 0.719. Similarly, Anatomical Therapeutic Chemical (ATC) codes were used to label drug pairs, and if a drug pair shares an ATC code, it was deemed as a positive sample. For anatomical, therapeutic, pharmacological, and chemical categories, the numbers of positive samples are 25 287, 7180, 3458, and 1146, respectively. Based on the ATC category data, Fig. 3B shows the AUC values for *R* and $\hat{R}$, respectively, and the AUC values for $\hat{R}$ are obviously higher than those for *R*. Apparently, the introduction of predicted frequency scores can verify that drugs with similar side effects may share targets or ATC codes.

Further, we tested the interpretability of drug embeddings and side effect embeddings. These embeddings were obtained by the NRFSE and Galeano’s model, respectively, which were trained with all available side effect frequencies in *R* and were used for calculating cosine similarity of drugs and effects. Likewise, for each drug pair, we labeled it as a positive or negative sample according to whether the two drugs have a shared target or ATC code. Figure 3C and D shows the ROC curves and the AUC bar charts for drug targets and ATC category data, respectively. As to drug targets, AUC of NRFSE is 0.732, which is 14.02% higher than that of Galeano’s model. For ATC category data, the two methods achieved similar performance. For each side effect pair, we treated it as positive or negative according to whether the two side effects have a shared MedDRA hierarchical terminology. For SOC, HLGT, and HLT, the numbers of positive samples are 56 170, 12 342, and 2270, respectively. Figure 3E shows the AUC bar charts for NRFSE and Galeano’s model. As to side effect embeddings of NRFSE, AUC values on SOC, HLGT, and HLT are 0.744, 0.833, and 0.892, which are 22.57%, 32.01%, and 28.53% better than those of Galeano’s model, respectively. Notably, we used MedDRA information for training NRFSE and the resulted side effect embeddings are biased for MedDRA information. As a contrast, we removed MedDRA information when training NRFSE and the resulted performance decreased dramatically, as shown in Fig. 3E. These results suggest that the embeddings obtained by NRFSE can better describe the target characteristics of drugs and the clinical semantic characteristics of side effects.

### 3.3 Model ablation analysis and sensitivity analysis

**Figure 4.**
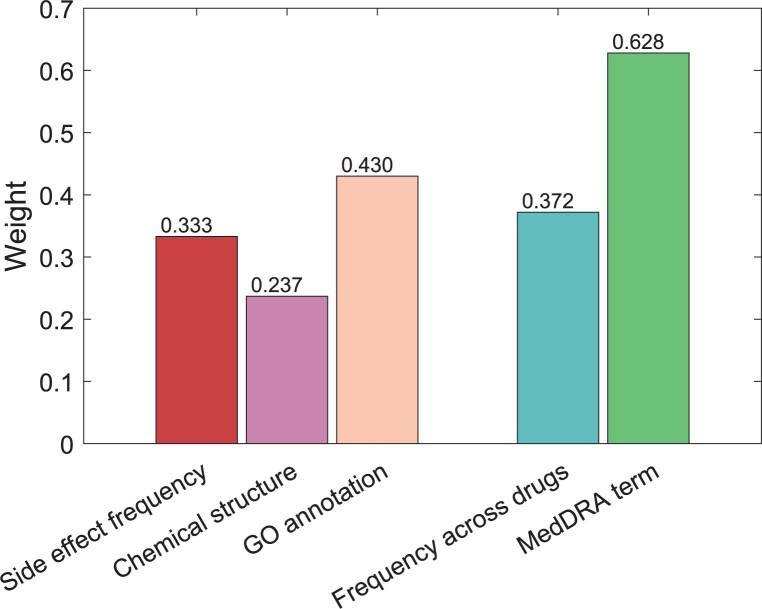
The weights for multiviews obtained by NRFSE.

Here, we performed a warm start-based ablation experiment on the three attributes of drugs and two attributes of effects, i.e. removing one attribute at a time, and then using the remaining information to perform a 10-fold CV under CV1 setting. As shown in Table 3, each of the attributes is valid in the prediction model, that is, the performance of the model lacking one attribute was worse than the model using all the attributes. Interestingly, our model can adaptively determine the optimal weights of attributes (shown in Fig. 4), and the relative sizes of weights are consistent with the results of the ablation experiment. For drugs, GO annotation and chemical structure are the most important and least important features, and their PCC values decrease the most and the least. For side effects, the MedDRA term has more weight than the frequency across drugs, and PCC value of the MedDRA term decreases more. Besides, our method replaces the embeddings of the new drug or the new side effect with the weighted average of the embedding of its *k*_2_-nearest neighbors. Here, we removed the above operation and resulted in decreased performance for 10-fold CV under CV2 setting, i.e. AUC = 0.897, AUPR = 0.434, RMSE = 2.236, MAE = 2.083, and PCC = 0.426.

**Table 3.** Ablation analysis on the attributes of drugs and effects under CV1 setting.

	Removed attribute	AUC	AUPR	RMSE	MAE	PCC
Drug	Effect frequency	0.921	0.260	1.015	0.775	0.574
	Chemical structure	0.924	0.266	**1.002**	0.762	0.578
	GO annotation	0.922	0.262	**1.002**	**0.760**	0.570
Effect	Frequency across drugs	0.919	0.256	**1.002**	0.764	0.576
	MedDRA term	0.917	0.252	1.011	0.764	0.560
\	None	**0.926**	**0.272**	1.008	0.767	**0.582**

‘\’ indicates that there is no entity. The best results are in bold.

**Figure 5.**
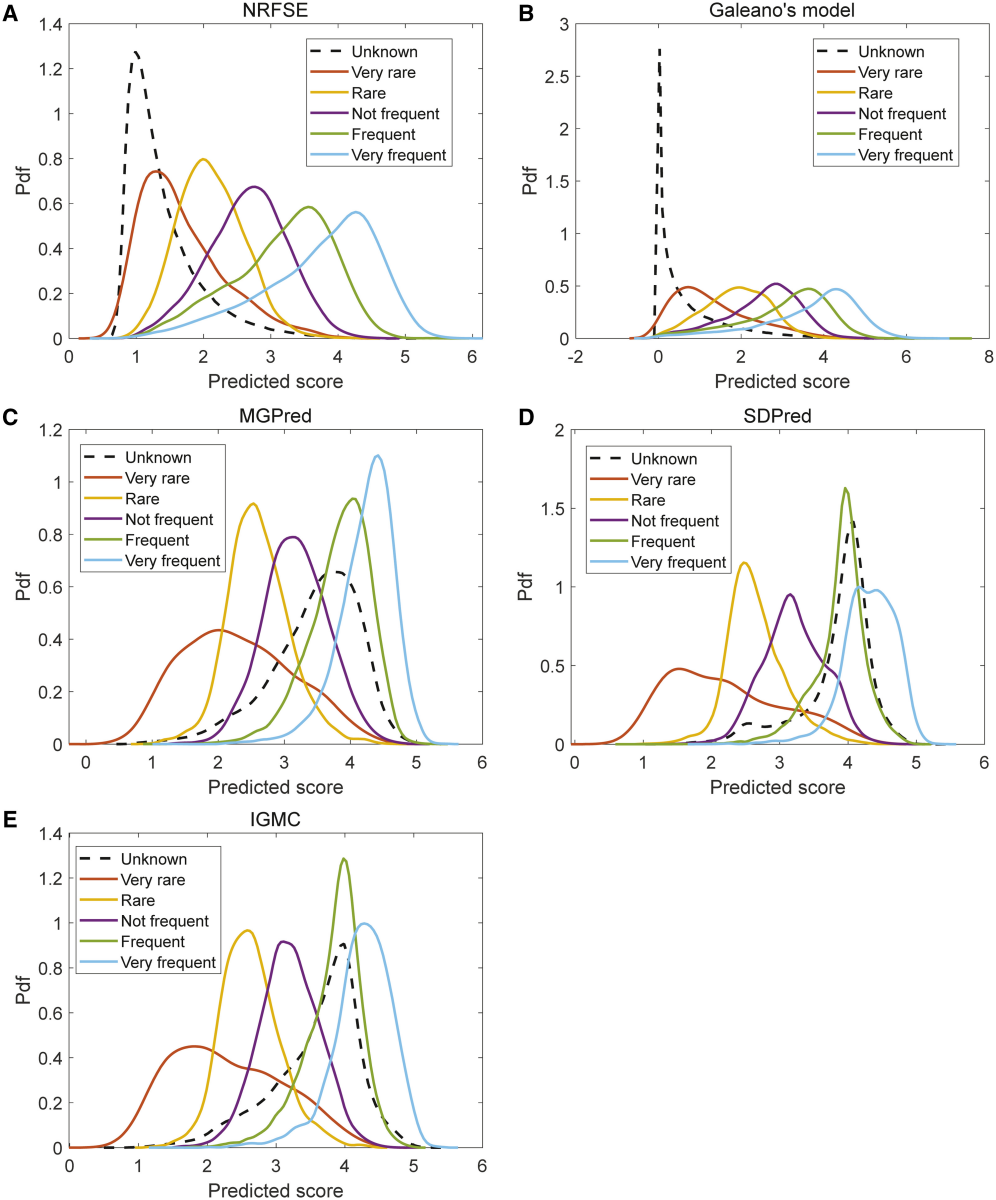
The probability density functions of predicted scores for each frequency class.

For unknown side effects and available known effects, we obtained their predicted scores from 1-fold and 10-fold CV under CV1 setting, respectively. Figure 5 shows the probability density functions of predicted scores from each frequency category for various methods, including NRFSE, Galeano’s model, MGPred, SDPred, and IGMC. Clearly, NRFSE and Galeano’s model can distinguish the distributions of predicted scores of unknown and known frequency terms. However, for other methods, the distribution of predicted scores of unknown frequency terms is close to that of predicted scores of frequent side effects (frequency = 4), which means that the unknown frequency terms tend to be classified as frequent category by these methods. A group of these methods, including MGPred, SDPred, and IGMC, seems to focus on the regression of frequency values alone and ignore the binary classification, i.e. the identification of true from false side effects. In fact, both are important in the problem of predicting frequencies of drug side effects, as initially formulated by Galeano *et al.* (2020).

**Figure 6.**
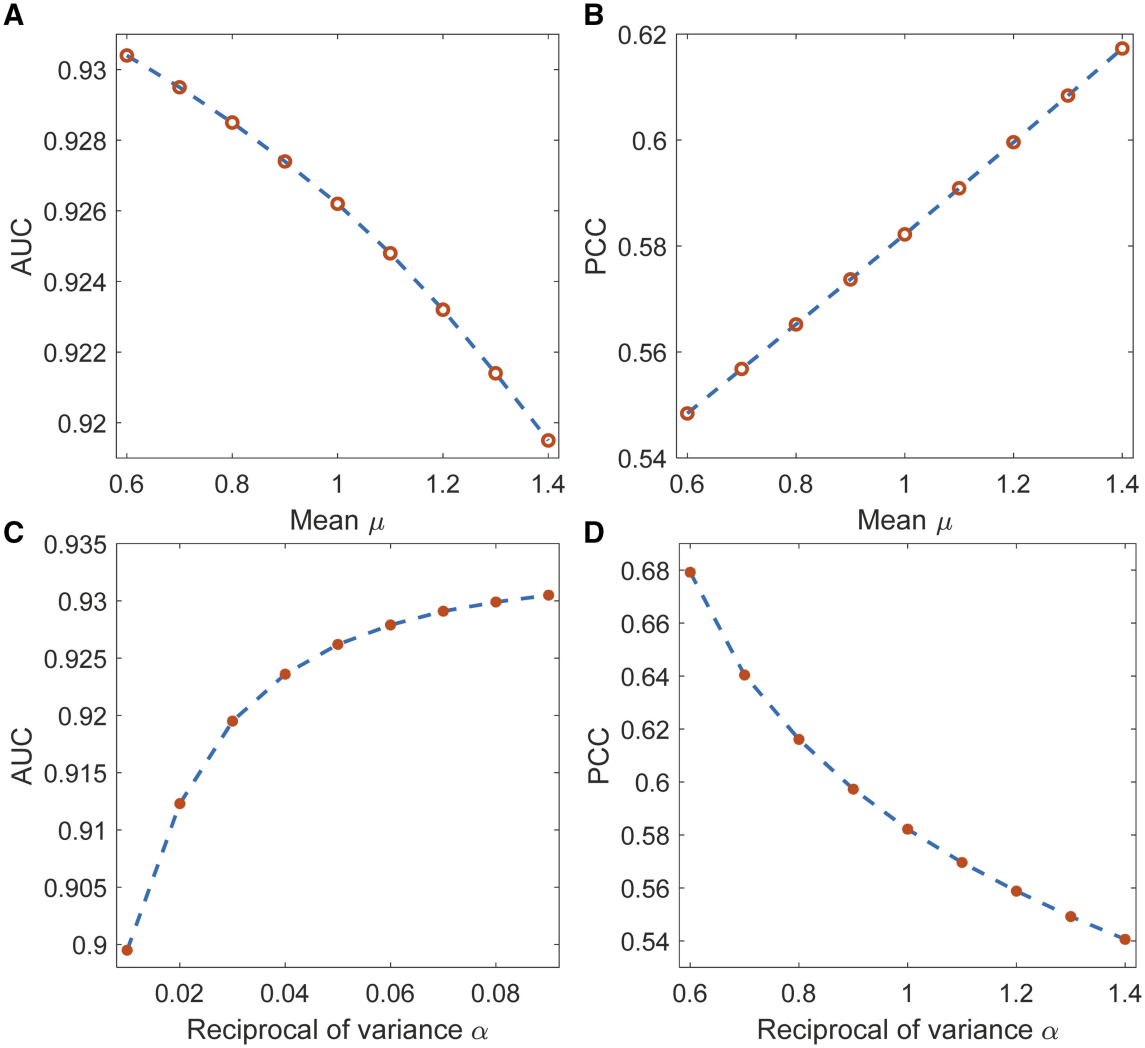
The trends of AUC and PCC with respect to mean *μ* and reciprocal of variance *α* for the distribution of predicted scores of unknown side effects.

Besides, our model NRFSE sets the predicted scores of the unknown side effects to satisfy the Gaussian distribution, the mean, and variance of which affect the interval between the distribution and the predicted score distribution of very rare effects, and the larger the interval when the mean *μ* is smaller or the reciprocal of variance *α* is larger. We performed sensitivity analysis on *μ* and *α* to investigate the relationship between the interval and prediction performance. Based on the 10-fold CV under CV1 setting, Figure 6A and B shows that the AUC value decreases monotonically with the *μ* value, while the PCC value increases gradually. Regarding the *α* value, the AUC value increases monotonically while the PCC value decreases gradually (Fig. 6C and D). These results show that the larger the interval, the stronger the model NRFSE’s ability to distinguish between unknown effects and known frequency effects, i.e. the higher AUC value, while the weaker the model’s ability to fit known frequency effects, i.e. the lower PCC value. Therefore, we set *μ *= 1 and α=0.05 to balance AUC value and PCC value. The sensitivity analysis results on other hyperparameters are shown in Supplementary Figs S1–S6.

### 3.4 Case studies

We used all known items in the frequency matrix *R* as the training set to predict the frequencies of all unknown items in *R*, and then took the top 10 side effects with higher scores for each drug. Table 4 lists the predicted results for four drugs, including alprostadil, diazepam, foscarnet, and conivaptan. As to alprostadil, diazepam, and foscarnet, each of them had less than 10 known side effects in the training set, but for their top 10 predicted side effects, 9, 10, and 8 were confirmed by Comparative toxicogenomics database (CTD, Davis *et al.* 2021) or SIDER respectively. Especially, alprostadil is known to cause hemodynamic instability, leading to hypotension/hypertension (Augustin *et al.* 2013). Diazepam increases anticholinergic adverse effects such as dry mouth, constipation, and blurred vision (Conti and Pinder 1979). Foscarnet may cause acute kidney injury, which is accompanied by increased serum creatinine (Navarro *et al.* 1996). Conivaptan had twenty available side effects in the training set. For the top 10 ranks of predictive side effects, five effects including nausea, pain, pruritus, vomiting, and diarrhea are confirmed by SIDER. Besides, conivaptan may have a serious effect of hypokalemia (Zeltser and Steinvil 2010).

**Table 4.** The top 10 ranks of predictive side effects for four drugs.

Drug (DrugBank ID)	Side effect (MedDRA code)
Alprostadil (DB00770)	Nausea (10028813)^a^; hypotension (10021097)^b^; headache (10019211)^a^; vomiting (10047700)^b^; hypertension (10020772)^a^; diarrhea (10012735)^a^; dermatitis (10012431); rash (10037844)^a^; tachycardia (10043071)^a^; pain (10033371)^b^
Diazepam (DB00829)	Nausea (10028813)^b^; headache (10019211)^a^; dizziness (10013573)^a^; fatigue (10016256)^a^; vomiting (10047700)^b^; dry mouth (10013781)^a^; diarrhea (10012735)^a^; constipation (10010774)^a^; dyspepsia (10013946)^a^; vision blurred (10047513)^b^
Foscarnet (DB00529)	Hyperglycemia (10020635); blood creatinine increased (10005483)^a^; hyponatremia (10021036)^b^; thrombocytopenia (10043554)^a^; hyperkalemia (10020646); leukopenia (10024384)^a^; dehydration (10012174)^a^; diarrhea (10012735)^a^; body temperature increased (10005911)^b^; neutropenia (10029354)^b^
Conivaptan (DB00872)	Nausea (10028813)^a^; pain (10033371)^a^; injection site reaction (10022095); hypokalemia (10021015); pruritus (10037087)^a^; vomiting (10047700)^a^; asthenia (10003549); diarrhea (10012735)^a^; injection site pain (10022086); blood creatinine increased (10005483)

a Side effect which is verified by SIDER.

b Side effect which is verified by CTD.

## 4 Conclusion

In this article, we developed a NRFSE for predicting frequencies of drug side effects. NRFSE has the following advantages over other state-of-the-art approaches. First, we characterize the distribution of predicted scores for unknown drug–side effect pairs by Gaussian distribution, in which the appropriate mean and variance are selected to balance the performances in AUC and PCC indicators. Second, the multiview data of drugs and side effects are integrated by multiview neighborhood regularization, in which the optimal weight for each view is adaptively determined. Third, the model puts the emphasis on the local structure of the drug–side effect frequency data, by taking advantage of *k*-nearest neighbors of drugs and side effects. Specifically, the model uses nearest neighbors to create neighborhoods of drugs and side effects, and refine embeddings of new drugs and new side effects. Compared to using all similar neighbors, the manner of nearest neighbors gets more accurate results by avoiding noisy information.

The performance of NRFSE was validated by 10-fold CV on one benchmark dataset. Clearly, in the comparative study, NRFSE showed better overall prediction accuracy than other methods. Besides, the prediction on an independent test set of post-marketing side effects illustrate that NRFSE could correctly identify their frequencies. At last, NRFSE obtained biologically significant latent signatures which could better describe the characteristics of drug targets and regulatory activities of side effects. NRFSE can serve as a tool to identify frequencies of drug side effects which can be used to guide randomized controlled trails. Furthermore, the multiview neighborhood regularization can be applied to other research fields such as drug repositioning (Zhang *et al.* 2020), drug–target interaction prediction (Liu *et al.* 2016), and drug–target binding affinity prediction (Li *et al.* 2022) by incorporating multiview data for drugs and targets.

## Supplementary Material

btad532_Supplementary_Data
