# Supplementary material for: A neighborhood-regularization method leveraging multiview data for predicting the frequency of drug–side effects
Source: Bioinformatics. 2023 Aug 30;39(9):btad532. doi: 10.1093/bioinformatics/btad532 (PMC10491955; doi:10.1093/bioinformatics/btad532)
Supplement: btad532_Supplementary_Data [file btad532_supplementary_data.pdf]

A neighborhood-regularization method leveraging multi-view data for predicting the frequency of drug side effects

Lin Wang*, Chenhao Sun, Xianyu Xu, Jia Li and Wenjuan Zhang*

1. Solving algorithm for the optimization model

We propose the following optimization model for predicting the frequency of drug side effects:

$$J(U,V,w_p,h_q)=\frac{1}{2}\parallel I^{\Omega}\odot(R-UV)\parallel_F^2+\frac{\alpha}{2}\parallel I^{o}\odot(UV-\mu E)\parallel_F^2$$

$$+\frac{\beta}{2}tr\left(U^T\sum_{p=1}^{x}{w_p}^{\delta}L_d^pU\right)+\frac{\gamma}{2}tr\left(V\sum_{q=1}^{y}{h_q}^{\delta}L_e^qV^T\right)$$

$$\text{s.t. } U\geq 0, V\geq 0$$

$$\sum_{p=1}^{x}w_p=1,\ w_p>0$$

$$\sum_{q=1}^{y}h_q=1,\ h_q>0.$$

We use an iterative update algorithm to solve our model. Specifically, w_p (p=1,…,x) and h_q (q=1,…y) are first fixed, and the multiplicative update procedure is applied to update U and V. The details are shown as follows:

$$\frac{\partial J}{\partial U}=-\left(I^{\Omega}\odot(R-UV)\right)V^T+\alpha(I^{o}\odot(UV-\mu E))V^T+\beta\sum_{p=1}^{x}{w_p}^{\delta}L_d^pU$$

$$=-RV^T+(I^{\Omega}\odot(UV))V^T+\alpha(I^{o}\odot(UV))V^T-\alpha\mu I^{o}V^T+\beta\left(\sum_{p=1}^{x}{w_p}^{\delta}\left(D_d^p+\widetilde{D}_d^p\right)\right)U-$$

$$\beta\left(\sum_{p=1}^{x}{w_p}^{\delta}\left(\tilde{A}_d^p+{\tilde{A}_d^{p}}^{T}\right)\right)U$$

$$=-(R+\alpha\mu I^{o})V^T-\beta\left(\sum_{p=1}^{x}{w_p}^{\delta}\left(\tilde{A}_d^p+{\tilde{A}_d^{p}}^{T}\right)\right)U+(I^{\Omega}\odot(UV)+\alpha I^{o}\odot(UV))V^T+$$

$$\beta\left(\sum_{p=1}^{x}{w_p}^{\delta}\left(D_d^p+\widetilde{D}_d^p\right)\right)U$$

$$\frac{\partial J}{\partial V}=-U^T\left(I^{\Omega}\odot(R-UV)\right)+\alpha U^T(I^{o}\odot(UV-\mu E))+\gamma V\sum_{q=1}^{y}{h_q}^{\delta}L_e^q$$

$$=-U^TR+U^T(I^{\Omega}\odot(UV))+\alpha U^T(I^{o}\odot(UV))-\alpha\mu I^{o}U^T+\gamma V\left(\sum_{q=1}^{y}{h_q}^{\delta}\left(D_e^q+\widetilde{D}_e^q\right)\right)-$$

$$\gamma V\left(\sum_{q=1}^{y}{h_q}^{\delta}\left(\tilde{A}_e^q+{\tilde{A}_e^{q}}^{\top}\right)\right)$$

$$=-U^T(R+\alpha\mu I^{o})-\gamma V\left(\sum_{q=1}^{y}{h_q}^{\delta}\left(\tilde{A}_e^q+{\tilde{A}_e^{q}}^{\top}\right)\right)+U^T(I^{\Omega}\odot(UV)+\alpha I^{o}\odot(UV))+$$

$$\gamma V\left(\sum_{q=1}^{y}{h_q}^{\delta}\left(D_e^q+\widetilde{D}_e^q\right)\right)$$

According to Karush-Kuhn-Tucker (KKT) dual complementarity condition, $\left(\frac{\partial J}{\partial U}\right)_{ij} U_{ij}$=0, $\left(\frac{\partial J}{\partial V}\right)_{ij} V_{ij}$=0. So the update formulas for *U* and *V* are designed as follows:

$$U \leftarrow U_0 \odot \frac{(R+\alpha\mu I^o)V_0^T + \beta(\sum_{p=1}^{x} w_p{}^{\delta}(\tilde{A}_d^p + \tilde{A}_d^{p\top}))U_0}{\left(I^{\Omega} \odot (U_0V_0) + \alpha I^o \odot (U_0V_0)\right)V_0^T + \beta(\sum_{p=1}^{x} w_p{}^{\delta}(D_d^p + \widetilde{D}_d^p))U_0}$$

$$V \leftarrow V_0 \odot \frac{U^\top(R+\alpha\mu I^o) + \gamma V_0(\sum_{q=1}^{y} h_q{}^{\delta}(\tilde{A}_e^q + \tilde{A}_e^{q\top}))}{U^\top(I^{\Omega} \odot (UV_0) + \alpha I^o \odot (UV_0)) + \gamma V_0(\sum_{q=1}^{y} h_q{}^{\delta}(D_e^q + \widetilde{D}_e^q))}$$

where U_0 and V_0 represent the matrices before updating, and U and V are the matrices after updating.

Then, we fix *U* and *V*, and the optimization for w_p (*p*=1,…,*x*) and h_q (*q*=1,…*y*) is shown as follows.

$$\min_{w_p,h_q} \frac{\beta}{2} tr\left(U^T \sum_{p=1}^{x} w_p{}^{\delta} L_d^p U\right) + \frac{\gamma}{2} tr\left(V \sum_{q=1}^{y} h_q{}^{\delta} L_e^q V^T\right)$$

$$s.t.\ \sum_{p=1}^{x} w_p = 1,\ w_p \geq 0$$

$$\sum_{q=1}^{y} h_q = 1,\ h_q \geq 0.$$

Since w_p and h_q are independent, we solve their optimal solutions, respectively. The Lagrange function for w_p is defined as follows

$$L(w_p, \lambda) = tr(U^T \sum_{p=1}^{x} w_p{}^{\delta} L_d^p U) - \lambda(\sum_{p=1}^{x} w_p - 1).$$

$$\frac{\partial L}{\partial w_p} = \delta tr(U^T L_d^p U) w_p{}^{\delta-1} - \lambda \text{ for } p=1,\ldots,x.$$

With $\frac{\partial L}{\partial w_p} = 0$, we can conclude $\lambda = \delta tr(U^T L_d^p U) w_p{}^{\delta-1}$, i.e. $w_p = (\frac{\lambda}{\delta tr(U^T L_d^p U)})^{\frac{1}{\delta-1}}$.

Because of $\sum_{p=1}^{x} w_p = 1$, we can obtain $\sum_{p=1}^{x}(\frac{\lambda}{\delta tr(U^T L_d^p U)})^{\frac{1}{\delta-1}} = 1$, i.e. $(\frac{\lambda}{\delta})^{\frac{1}{\delta-1}} = \frac{1}{\sum_{p=1}^{x}(\frac{1}{tr(U^T L_d^p U)})^{\frac{1}{\delta-1}}}$. So $w_p = \frac{(\frac{1}{tr(U^T L_d^p U)})^{\frac{1}{\delta-1}}}{\sum_{p=1}^{x}(\frac{1}{tr(U^T L_d^p U)})^{\frac{1}{\delta-1}}}$. Similarly, we can get optimal solution for h_q with $h_q = \frac{(\frac{1}{tr(V L_e^q V^T)})^{\frac{1}{\delta-1}}}{\sum_{q=1}^{y}(\frac{1}{tr(V L_e^q V^T)})^{\frac{1}{\delta-1}}}$.

2. Sensitivity analysis of hyperparameters

Here, we present the sensitivity analysis results of the remaining hyperparameters in the NRFSE under the CV1 setting, including the regularization coefficients for drugs and side effects (β and γ), the latent feature dimension for drugs and side effects (k), the number of neighbors for drugs and side effects (k_1) and the number of neighbors for new drugs and new side effects (k_2). We conducted 10-fold cross validation for each parameter under the CV1 setting, and then calculated five metrics for each fold,

such as AUC, AUPR, MAE, RMSE and PCC, and drew a boxplot of the 10 fold results for each metric ('+' represents the mean value). Among the five metrics, AUC and AUPR represent the predictive ability of the NRFSE to predict drug-side effect associations, while MAE, RMSE, and PCC represent the predictive ability of the NRFSE to predict drug-side effect frequencies. It is worth noting that when testing each hyperparameter, we fixed other hyperparameters as default values, where the default values are $\beta = 2$, $\gamma = 2$, $k = 200$, $k_1 = 20$, and $k_2 = 10$。

1) Regularization coefficients for drugs and side effects

We selected β and γ values from {0, 1, 2, 3, 4} respectively, where 0 represents no regularization constraint, and the larger the value, the stronger the regularization constraint. Fig. S1 and S2 respectively show the sensitivity analysis results of β and γ. They illustrate that the results with regularization constraints are better than those without. Although the association prediction performance of $\beta = 1$ and $\gamma = 1$ is better than that of $\beta = 2$ and $\gamma = 2$, the frequency prediction performance is worse than that of $\beta = 2$ and $\gamma = 2$. The performance of $\beta = 2$ and $\gamma = 2$ is generally better than those of $\beta = 3$, $\beta = 4$ and $\gamma = 3$, $\gamma = 4$.

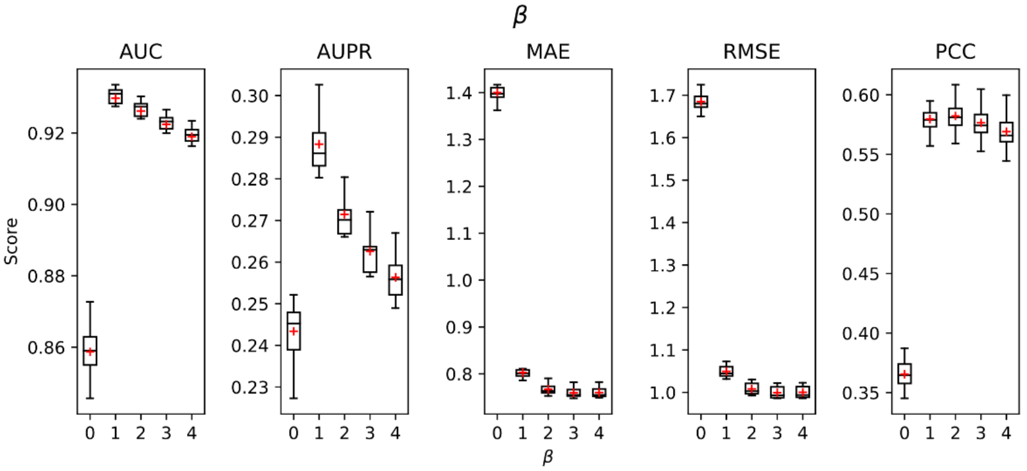

Fig. S1. The sensitivity analysis for regularization coefficient β for drugs

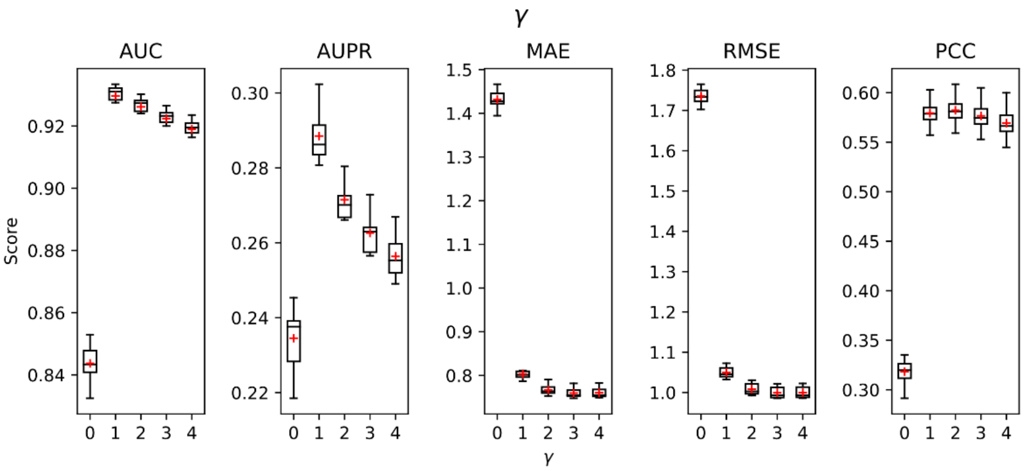

Fig. S2. The sensitivity analysis for regularization coefficient γ for side effects

2) Latent feature dimension of drugs and side effects

We selected k from {100, 150, 200, 250, 300}. Fig. S3 shows the results of the sensitivity analysis for k. It illustrates that PCC value with $k = 200$ are almost the same as that with $k = 250$ or $k = 300$, while AUPR value is generally better than that with $k = 250$ or $k = 300$. The overall performance of the NRFSE with $k = 200$ is better than those of $k = 100$ and $k = 150$.

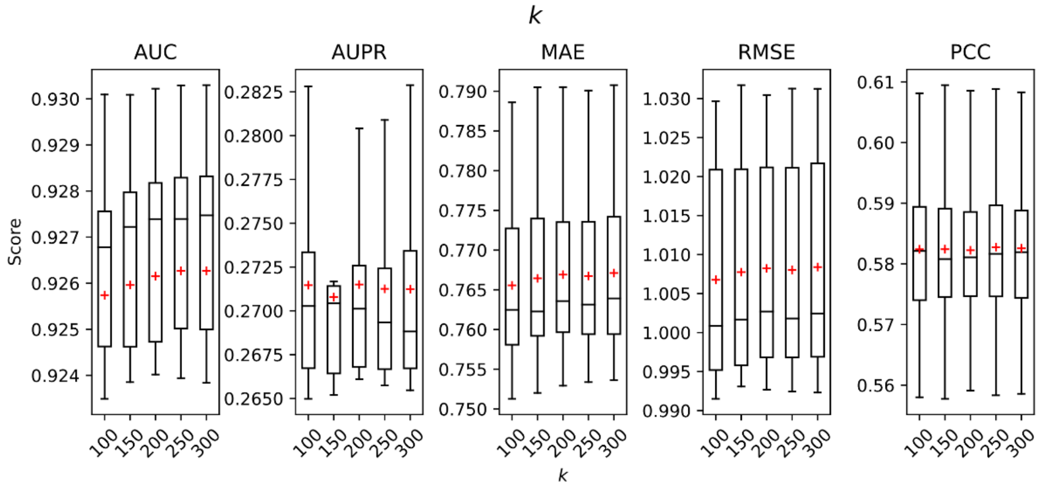

Fig. S3. The sensitivity analysis for the latent feature dimension k of drugs and side effects.

3) k_1-nearest neighbors of drugs and side effects

We selected k_1 from {5, 10, 15, 20, 25}. Fig. S4 shows the sensitivity analysis results of k_1. It illustrates that with the increase of k_1, NRFSE tends to decrease the performance of association prediction and improve the performance of frequency prediction. Therefore, $k_1 = 20$ was selected to balance the performances of association prediction and frequency prediction of NRFSE.

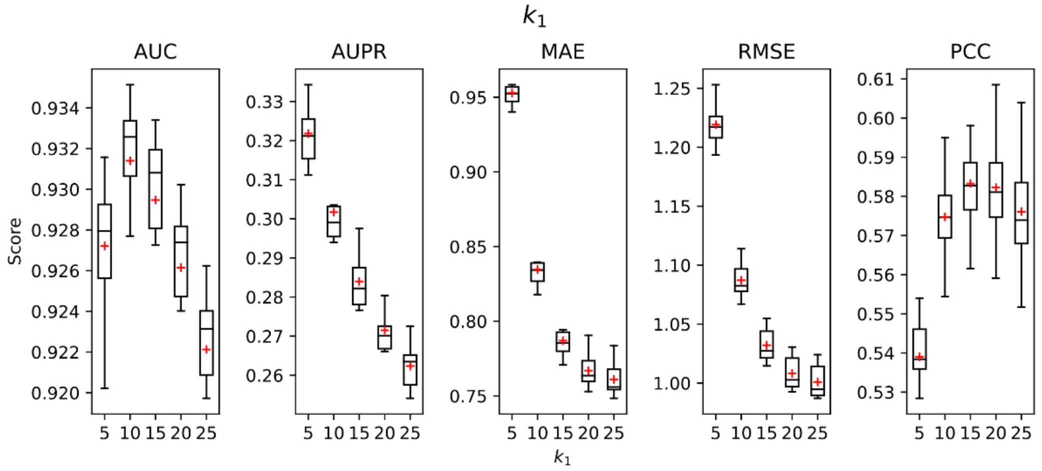

Fig. S4. The sensitivity analysis for k_1-nearest neighbors for drugs and side effects.

4) k_2-nearest neighbors of new drugs and new side effects

We selected k_2 from {1, 5, 10, 15, 20}. For new drugs or new side effects, the embedding directly learned by the model is inaccurate due to the lack of the

corresponding drug-side effect frequency terms, so we use the k_2-nearest neighbors of the corresponding drug or side effect to obtain the embedding by means of weighted average. Fig. S5 and S6 show the sensitivity analysis under the settings of CV1 and CV2, respectively. Because under CV1 setting, there are rarely new drugs or new side effects, the value of k_2 will not have an impact on the prediction results, as shown in Fig. S5. However, in the case of new drugs or new side effects, that is, under CV2 setting, the overall prediction performance of NRFSE first increases and then decreases, as shown in Fig. S6. It illustrates that when $k_2 = 10$, the association prediction performance of NRFSE is better than other options, and the frequency prediction performance is also excellent.

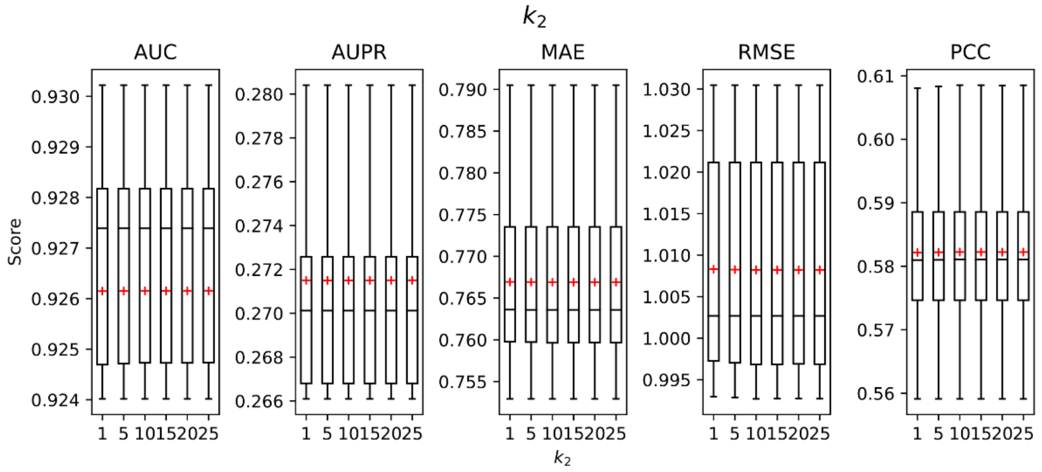

Fig. S5. The sensitivity analysis for k_2-nearest neighbors for new drugs and new side effects under CV1 setting.

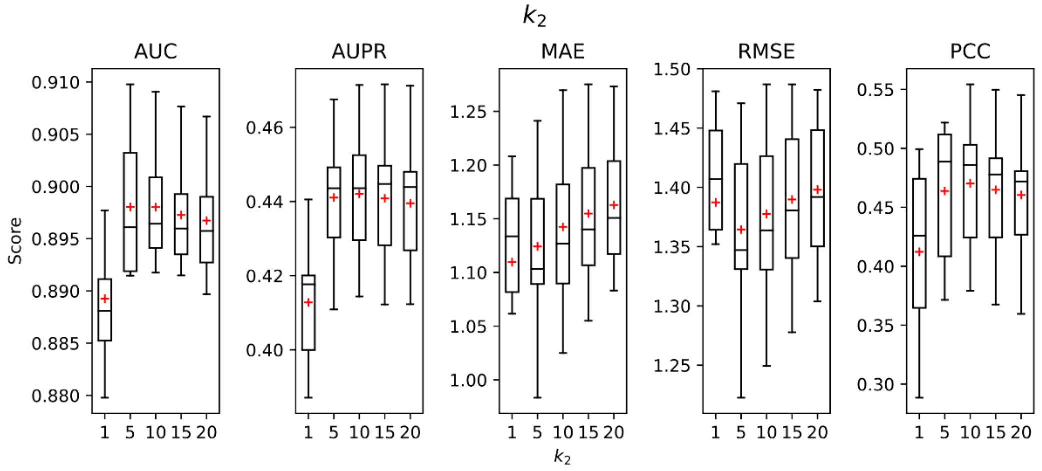

Fig. S6. The sensitivity analysis for k_2-nearest neighbors for new drugs and new side effects under CV2 setting.
